# *SULT* and *UGT* Genetic Variants Modulate Side Effect Profiles in South African Breast Cancer Patients Treated with Tamoxifen

**DOI:** 10.3390/genes17030252

**Published:** 2026-02-24

**Authors:** Bianca Kruger, Emile Chimusa, Aron Abera, Jesmika Singh, Delva Shamley, Collet Dandara

**Affiliations:** 1 Platform for Pharmacogenomics Research and Translation (PREMED), South African Medical Research Council, Cape Town 7700, South Africa; krgbia001@myuct.ac.za (B.K.); sngjes003@myuct.ac.za (J.S.); 2 Pharmacogenomics and Drug Metabolism Research Group, Division of Human Genetics, Department of Pathology, Institute of Infectious Diseases and Molecular Medicine, Faculty of Health Sciences, University of Cape Town, Cape Town 7700, South Africa; 3 Bioinformatic and Multi-Omics Data Science Group, Faculty of Science and Environment, Northumbria University, Newcastle NE1 8ST, UK; emile.chimusa@northumbria.ac.uk; 4 Inqaba Biotechnical Industries, Pretoria 0002, South Africa; aron.abera@inqababiotec.ac.za; 5 Division of Clinical Anatomy and Biological Anthropology, Department of Human Biology, Faculty of Health Sciences, University of Cape Town, Cape Town 7700, South Africa; delva.shamley@uct.ac.za

**Keywords:** Africa, breast cancer, pharmacogenetics, side effects, sulfotransferases, tamoxifen, UDP-glucuronosyltransferases

## Abstract

**Background**: Tamoxifen remains the cornerstone of endocrine therapy for hormone receptor-positive breast cancer across Africa. Understanding the factors that influence tamoxifen tolerability is critical, as treatment-related side effects can reduce adherence and compromise therapeutic outcomes. Yet, the contribution of pharmacogenetic variation to tamoxifen-related toxicity remains poorly characterized in African populations. This study, therefore, investigated whether genetic variation in key pharmacogenes influences the risk of treatment-related side effects in a South African breast cancer cohort. **Methods**: A total of 166 women of Mixed and African Ancestry treated with 20 mg/day tamoxifen at Groote Schuur Hospital, South Africa, were included in the study. Genetic variation across 28 variants in nine pharmacogenes, including *CYP2D6*, *CYP3A4/5*, *UGT1A4*, *UGT2B7/15*, *SULT1A1/2*, and *SULT1E1*, was assessed using various genotyping methods. Associations between genetic and non-genetic factors and tamoxifen side effects were evaluated with logistic regression. **Results**: Over 70% of participants reported at least one treatment-related side effect. Overall side-effect burden was associated with *SULT1A1* copy number variation (*p* = 0.030) and *SULT1E1* rs3736599 (*p* = 0.042). Musculoskeletal complaints were the most common (40%) and were associated with *UGT2B7* rs7439366 (*p* = 0.040) and *CYP3A4* rs2242480 (*p* = 0.051). Gynecological symptoms affected more than 20% of participants and were linked to SULT1A2*2 (*p* = 0.050), *SULT1E1* rs3736599 (*p* = 0.016), and *UGT2B15* rs4148269 (*p* = 0.039). Hot flashes were frequent, affecting 33% of patients, but showed no clear pharmacogenetic associations. **Conclusions**: This study demonstrates that pharmacogenetic variation is associated with interindividual differences in treatment-related side effects, underscoring the need to expand research in African populations to better inform precision endocrine therapy.

## 1. Introduction

Breast cancer is the most prevalent cancer and the leading cause of cancer-related mortality among African women [1]. In sub-Saharan Africa, an estimated 133,520 new female breast cancer cases occurred in 2022, and in South Africa, an estimated 14,712 new breast cancer cases were diagnosed in 2022 [1]. More than half of breast cancer cases are estrogen receptor-positive (ER+) [2]. Tamoxifen remains the primary adjuvant endocrine therapy for ER+ breast cancer and is widely used across Africa, with reported usage ranging from 38% to 93% [3]. Although tamoxifen is highly effective in reducing recurrence and mortality [4], its clinical benefit is often compromised by treatment-related toxicities [5].

Tamoxifen can cause a spectrum of side effects, ranging from vasomotor and mood-related symptoms to more severe outcomes such as venous thromboembolism, stroke, and endometrial cancer [5]. More than 50% of African breast cancer patients experience side effects, with hot flashes being the most prevalent [6]. However, isolating tamoxifen-specific effects can be challenging, as many patients present with comorbidities and are exposed to polypharmacy, both of which may contribute to overall symptom burden [7]. These toxicities negatively impact quality of life and are a major contributor to non-adherence and treatment discontinuation [8,9], ultimately compromising survival outcomes [10]. Identifying patients at increased risk of treatment-related side effects is critical for guiding strategies to optimize therapeutic outcomes.

Tamoxifen is administered as a prodrug, requiring metabolic activation to potentiate its anti-estrogenic effects. The formation of tamoxifen metabolites, particularly endoxifen, is primarily mediated by the cytochrome P450 enzymes CYP2D6 and CYP3A4/5. Subsequent phase II metabolism via UDP-glucuronosyltransferase (UGT) and sulfotransferase (SULT) enzymes facilitates elimination of tamoxifen and its metabolites [11]. Genetic variation in tamoxifen-metabolizing enzymes is a major contributor to interindividual variability in tamoxifen response, as altered enzyme activity can influence metabolite concentrations [12,13,14].

Most pharmacogenetic research on tamoxifen has focused on survival outcomes. While important, comparatively few studies have investigated associations with treatment-related side effects. Furthermore, the limited evidence available is largely derived from European and Asian populations [15], making extrapolation to the genetically diverse African populations inappropriate [16]. Consequently, the genetic determinants of treatment-related side effects remain largely unexplored in African patients. Thus, this study aimed to characterize the relationship between genetic variation and the occurrence of side effects in a South African cohort of breast cancer patients receiving tamoxifen.

## 2. Materials and Methods

### 2.1. Study Design, Setting, and Participants

This cross-sectional study evaluated the association between pharmacogenetic variation and treatment-related side effects in breast cancer patients receiving tamoxifen and other co-administered medications, using data extracted from clinical records. The study represents a single-center, retrospective convenience cohort derived from patients treated at Groote Schuur Hospital in Cape Town, South Africa. Participant recruitment and sample collection procedures have previously been described [17]. Briefly, women attending routine follow-up appointments for breast cancer between 2013 and 2018 were recruited as part of a broader study investigating genetic contributors to shoulder pain and disability among breast cancer patients. DNA samples were collected at enrollment, and relevant clinical information was obtained retrospectively from hospital records. The broader study was approved by the University of Cape Town Human Research Ethics Committee (Ref: 312/2012), and all participants provided written informed consent. Participants self-identified as being Mixed Ancestry, African Ancestry, or European Ancestry. The Mixed Ancestry population reflects extensive genetic admixture, with ancestral contributions from indigenous Africans, European settlers, and migrant Asian populations [18].

DNA samples were available from 268 participants in the parent cohort for genetic characterization. Hospital records were reviewed for clinical information, including reports of treatment-related side effects. Of these, 166 participants met the eligibility criteria for the present analysis. Eligibility required: (i) a diagnosis of primary breast carcinoma; (ii) self-reported Mixed or African Ancestry; (iii) tamoxifen prescribed at the standard dose of 20 mg/day; and (iv) tamoxifen exposure for ≥4 months, corresponding to the approximate time required to achieve steady-state serum concentrations of tamoxifen and its active metabolites [19]. Participants of European Ancestry were excluded due to their small sample size (*n* = 10) and to maintain focus on populations of African Ancestry.

No formal a priori sample size calculation was performed, as the sample size was constrained by the retrospective nature of the project. Ethical approval for the present analysis was granted by the University of Cape Town Human Research Ethics Committee (Ref: 795/2020).

### 2.2. Study Endpoint

The study measured the outcome, or endpoint, which was the presence or absence of treatment-related side effects. Side effects were classified as follows: vasomotor events in the form of hot flashes; musculoskeletal events, comprising joint pain, leg cramps and myalgia; gynecological events, such as vaginal discharge, dryness, itchiness, and abnormal menstruation; neurological events, including paresthesia, mood swings and depression; thromboembolic events, such as thrombosis and stroke; and endometrial events, including endometrial hyperplasia or endometrial cancer.

### 2.3. Gene Selection and Genetic Characterization

Nine pharmacogenes known to be involved in the tamoxifen metabolic pathway (*CYP2D6*, *CYP3A4*, *CYP3A5*, *UGT1A4*, *UGT2B7*, *UGT2B15*, *SULT1A1*, *SULT1A2*, and *SULT1E1*) were characterized based on evidence from the Pharmacogenomics Knowledge Base [11]. Single-nucleotide polymorphisms (SNPs) with a minor allele frequency (MAF) > 0.05 in African (AFR) populations were identified from the Ensembl database www.ensembl.org (accessed on 9 September 2020), supplemented by targeted literature searches. This exploratory, population-focused approach prioritized variants most relevant to African genetic diversity.

DNA was extracted from whole blood samples and stored as previously described [17]. Genotyping was performed using a custom panel optimized by Inqaba Biotec (Pretoria, South Africa) for analysis on the MassARRAY^®^ System (Agena Bioscience, San Diego, CA, USA). *SULT1A1* variation was characterized via Sanger sequencing. Primers flanking seven *SULT1A1* SNPs (rs4149393, rs4149394, rs1042028, rs1801030, rs28374453, rs6839 and rs1042157) were designed, and polymerase chain reaction (PCR) was performed in 25 µL reaction mixtures containing Green GoTaq^®^ Flexi Reaction Buffer (Promega Corporation, Madison, WI, USA), 0.2 mM dNTPs (Promega Corporation), 2 mM magnesium chloride (Promega Corporation), 0.4 µM of each primer (forward: 5′-CTGTCCTCCAGTGATCCTCTAG-3′; reverse: 5′-ATAGGAGCCAAGCCCAGCTCAT-3′), 0.65 U GoTaq^®^ G2 Flexi DNA Polymerase (Promega Corporation), 10–50 ng genomic DNA, and nuclease-free water. PCR was carried out in a SimpliAmp™ Thermal Cycler (Applied Biosystems, Carlsbad, CA, USA) under the following cycling conditions: 95 °C for 3 min; 35 cycles of 95 °C for 30 s, 64 °C for 30 s, and 72 °C for 1 min; and a final extension at 72 °C for 5 min. Post-PCR cleanup for Sanger sequencing used 4 U Exonuclease I and 1 U FastAP™ Thermosensitive Alkaline Phosphatase (Thermo Fisher Scientific, Waltham, MA, USA) in a 20 µL reaction, incubated at 37 °C for 1 h, and inactivated at 75 °C for 15 min. Sequencing reactions were set up in 10 µL volumes containing BigDye™ Terminator v3.1 Ready Reaction Mix (Applied Biosystems), BigDye™ Terminator v3.1 Sequencing Buffer (Applied Biosystems), and 1 µM of forward or reverse primer. Cycling conditions were: 98 °C for 5 min; 25 cycles of 96 °C for 30 s, 50 °C for 15 s, and 60 °C for 4 min. Capillary electrophoresis was performed on an ABI 3730xl DNA Analyzer (Applied Biosystems).

Copy number variation (CNV) in *CYP2D6* and *SULT1A1* was assessed using the VeriDose^®^ *CYP2D6* CNV Panel (Agena Bioscience) and *SULT1A1* TaqMan^®^ Copy Number Assay (Hs04461762_cn, Applied Biosystems), respectively. For *SULT1A1* CNV detection, a 10 μL reaction contained 1 ng genomic DNA, TaqPath™ ProAmp™ Master Mix (Applied Biosystems), TaqMan CNV assay, and TaqMan™ Copy Number Reference Assay (human RNase P, Applied Biosystems). Reactions were run in duplicate on a QuantStudio™ 7 Flex Real-Time PCR System (Applied Biosystems) with cycling at 95 °C for 10 min, followed by 40 cycles of 95 °C for 15 s and 60 °C for 60 s. Cycle threshold (Ct) values were recorded using a manual threshold of 0.2 and an automatic baseline. CNV calls were generated using CopyCaller™ v2.1 (Applied Biosystems) via the comparative Ct method. No external calibrator sample was included; instead, the most common copy number in the dataset was set to three, consistent with the higher *SULT1A1* copy number commonly reported in African populations [20].

### 2.4. Statistical Analysis

Demographic and clinical characteristics were analyzed using STATA^®^ SE version 15.0. Categorical variables were summarized as counts and percentages (*n*, %), while continuous variables were reported as mean ± standard deviation (SD) or medians with interquartile ranges (IQR), depending on data distribution. Normality of continuous variables was assessed using the Shapiro–Wilk test. Hardy–Weinberg equilibrium and allele frequencies for each SNP were assessed using SHEsis http://shesisplus.bio-x.cn/SHEsis.html (accessed on 12 May 2025). Linkage disequilibrium (LD) between SNPs was evaluated in HaploView version 4.2.

Association analyses were conducted in R version 4.2.2 using an additive genetic model. Given the complexity of *CYP2D6* variation, *CYP2D6* genotypes were grouped into predicted phenotype categories: poor (PM), intermediate (IM), normal/extensive (EM), and ultrarapid (UM) metabolizers. Phenotypes were classified using CYP2D6 activity scores (AS) in accordance with Clinical Pharmacogenetics Implementation Consortium guidelines: PM (AS = 0), IM (0 < AS < 1.25), EM (1.25 ≤ AS ≤ 2.25), and UM (AS > 2.25) [21]. Analyses were restricted to SNPs with a MAF > 0.05 in the study cohort, resulting in 26 SNPs included in association testing; CYP2D6 phenotype and *SULT1A1* CNV were each treated as additional genetic predictors.

Associations with treatment-related side effects were evaluated using logistic regression, adjusting for ethnicity. Variables with *p* ≤ 0.10 in univariate analyses were carried forward into multivariate models. Missing genotype and clinical data were handled using category-based approaches that retained participants in the analysis without interpreting missing values. Statistical significance was defined as *p* < 0.05. To account for multiple testing, the Bonferroni correction was applied based on the 28 genetic variables included, yielding a corrected significance threshold of *p* < 0.002. Post hoc power calculations were conducted using R.

## 3. Results

### 3.1. Participant Characteristics

Participant demographic and clinical characteristics are summarized in Table 1. The cohort included 166 South African breast cancer patients, of whom 83.7% self-identified as Mixed Ancestry and 16.3% as African Ancestry. At diagnosis, 42% were postmenopausal, 40% were pre- or perimenopausal; menopausal status was unknown for the remainder. The median body mass index (BMI) was 29.5 kg/m^2^, and 39.2% reported a history of smoking. Over 90% of participants were prescribed concomitant medications alongside tamoxifen.

The mean age at diagnosis was 51.8 years (SD ± 10.3). Most participants (89.2%) were diagnosed with invasive ductal carcinoma (IDC), and 69.9% presented with tumor stage II or higher. Fifty-five patients (33.1%) transitioned to an alternative endocrine therapy during follow-up; 11.4% of these switches were attributed to treatment-related side effects, while the remainder were due to disease recurrence or clinician concern for progression.

**Figure 1:**
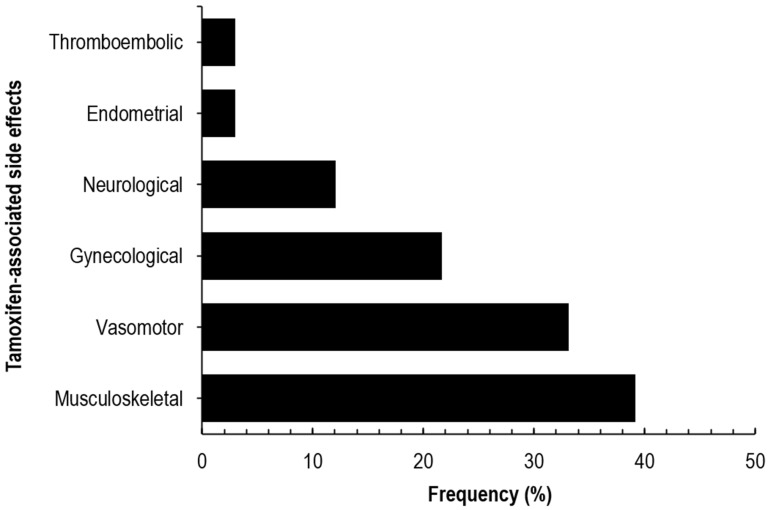
Prevalence of treatment-related side effects in South African breast cancer patients. Thromboembolic events include thrombosis and stroke. Endometrial events include endometrial hyperplasia or endometrial cancer. Neurological events include paresthesia, mood swings, and depression. Gynecological events include vaginal discharge, dryness, itchiness, and abnormal menstruation; Vasomotor events refer to hot flashes. Musculoskeletal events include joint pain, leg cramps, and myalgia.

Treatment-related side effects were common and reported among 121 participants (72.9%). Musculoskeletal symptoms were most frequent (Figure 1), affecting 39.2% of participants, followed by vasomotor (33.1%), gynecological (21.7%), neurological (12.0%), endometrial (3.0%), and thromboembolic symptoms (3.0%).

*SULT1A1* presented with unusually high copy numbers not previously reported, with one African-Ancestry participant carrying seven copies and two individuals (one in each ancestry group) carrying eight copies (Figure S1). Notably, there was no correlation between *SULT1A1* copy number and genotype.

### 3.2. Association of Clinical and Genetic Factors with Side Effect Profiles

Logistic regression analyses, adjusted for ethnicity, were performed for overall side effects followed by symptom-specific outcomes (Table 2). Symptom-specific analyses were restricted to categories reported by ≥20% of participants.

#### 3.2.1. Overall Side Effects

No variables reached statistical significance in univariate analysis; however, *SULT1A1* copy number, *SULT1E1* rs3736599, and IDC subtype demonstrated suggestive associations (*p* ≤ 0.1) and were included in the multivariate model. In this model, both genetic factors showed significant associations with increased side-effect risk. Participants with ≤3 *SULT1A1* copies had more than double the risk of experiencing side effects compared to those carrying ≥4 copies (odds ratio [OR] = 2.48; 95% confidence interval [CI] = 1.09–5.69; *p* = 0.030). Similarly, heterozygotes for *SULT1E1* rs3736599 showed increased risk of side effects compared with those homozygous for the reference allele (OR = 2.67; 95% CI = 1.08–7.38; *p* = 0.042). Neither association remained statistically significant after Bonferroni correction.

#### 3.2.2. Musculoskeletal-Associated Side Effects

Higher BMI showed a modest association with increased musculoskeletal symptoms in univariate analysis (OR = 1.06; 95% CI = 1.00–1.11; *p* = 0.047). Carriers of the *UGT2B7* rs7439366 variant allele had decreased odds of musculoskeletal symptoms, with the strongest effect observed among heterozygotes (OR = 0.26; 95% CI = 0.07–0.93; *p* = 0.040). Heterozygotes for *CYP3A4* rs2242480 also showed a borderline protective association in both univariate and multivariate models (OR = 0.35; 95% CI = 0.12–1.00; *p* = 0.051). None of these associations remained significant after Bonferroni correction.

#### 3.2.3. Vasomotor-Associated Side Effects (Hot Flashes)

In univariate analysis, individuals homozygous for *UGT1A4* rs11888492 showed a trend toward a reduced risk of hot flashes (OR = 0.24; 95% CI = 0.05–1.25; *p* = 0.091); however, this association did not persist in the multivariate model. *SULT1A1* rs4149393 was included in the models as a representative marker for rs4149393, rs4149394, and rs1042157 due to strong LD among these variants (D′ = 1; R^2^ = 0.9). None of these variants were significantly associated with hot flashes, and all three deviated from the Hardy–Weinberg equilibrium in the Mixed Ancestry group. In univariate analysis, BMI showed an inverse relationship with hot flashes (OR = 0.93; 95% CI = 0.88–0.99; *p* = 0.029). In multivariate analysis, none of the genetic variables remained significant. Smoking history was the only independent predictor, with smokers having over twice the odds of reporting hot flashes compared with non-smokers (OR = 2.74; 95% CI = 1.12–6.95; *p* = 0.029). The association was no longer significant following Bonferroni adjustment.

#### 3.2.4. Gynecological-Associated Side Effects

Heterozygosity for SULT1A2*2 (rs1059491 and rs1136703) and *SULT1E1* rs3736599 was associated with a 2-fold increased risk of gynecological symptoms. In multivariate analysis, *SULT1E1* rs3736599 remained significantly associated (OR = 2.87; 95% CI = 1.22–6.91; *p* = 0.016), while SULT1A2*2 showed a borderline significant effect with gynecological symptoms (OR = 2.22; 95% CI = 1.01–5.05; *p* = 0.050). In contrast, heterozygotes for *UGT2B15* rs4148269 had significantly reduced odds of gynecological symptoms (OR = 0.37; 95% CI = 0.14–0.93; *p* = 0.039). None of these associations remained significant after Bonferroni correction.

Prescription of co-medications was not significantly associated with overall or symptom-specific side effects (all *p* > 0.05). In particular, prescription of CYP2D6 inhibitors, reported in 22.3% of participants, was not associated with overall side effects or any specific side-effect category (all *p* > 0.05). CYP2D6-predicted phenotype was likewise not associated with overall or symptom-specific side effects (Table S1).

Post hoc power calculations indicated that, at α = 0.05, the study had approximately 58–70% power to detect ORs in the range of 2.5–2.8 for variants with MAF between 0.25 and 0.35. However, when applying the Bonferroni-adjusted significance threshold (α = 0.002), statistical power decreased to below 25% for most observed effect sizes.

## 4. Discussion

Treating breast cancer patients with tamoxifen remains the cornerstone of endocrine therapy for ER+ breast cancer across Africa, largely due to its affordability, broad accessibility, and suitability for women who are frequently diagnosed at younger, premenopausal ages [3,22]. Treatment options for premenopausal patients with ER+ disease are limited, making tamoxifen particularly critical in this setting. However, treatment-related side effects are common and can substantially reduce adherence, ultimately compromising therapeutic benefit [23]. Although pharmacogenetic associations with tamoxifen side effects have been reported in several studies [24,25,26,27], findings have been inconsistent across cohorts [6,28,29,30]. Prior work has focused almost exclusively on *CYP2D6*, with minimal representation of African populations. Given the high genetic diversity and unique allele frequency patterns across African ancestries, population-specific pharmacogenetic investigations are essential to improve the relevance and accuracy of predictive biomarkers in these settings [16].

In this study, we evaluated treatment-related side effects and their pharmacogenetic determinants in a South African cohort. We observed a high burden of side effects and identified novel associations involving phase II metabolizing enzymes, suggesting that variation beyond *CYP2D6* may also meaningfully influence tamoxifen tolerability.

Over 70% of participants experienced at least one side event, consistent with previous reports [24,28,31]. *SULT1A1* copy number emerged as a key determinant of overall side-effect burden: individuals with three or fewer copies had a higher likelihood of experiencing side effects compared with those carrying four or more copies. One study previously investigated the effects of *SULT1A1* CNV on tamoxifen-associated adverse reactions [32]. However, only a single participant in their cohort carried a copy number other than two, limiting the ability to draw meaningful conclusions and precluding comparison with our findings.

Prior studies have linked higher endoxifen levels to increased frequency or severity of side effects [24,33,34]. As SULT1A1 activity correlates with gene copy number [35], greater sulfation capacity may accelerate endoxifen clearance, thereby reducing metabolite accumulation and lowering toxicity risk. This could explain the protective effect observed in participants with higher copy numbers in our cohort. However, evidence remains mixed, as at least one study found no association between *SULT1A1* copy number and tamoxifen metabolite levels [36]. We also observed that heterozygotes for *SULT1E1* rs3736599 exhibited a higher proportion of side effects. This may similarly reflect altered endoxifen clearance, although the functional impact of this variant remains unclear. Studies to date provide conflicting evidence, suggesting that rs3736599 may either increase or decrease sulfation activity [37,38], and its role in tamoxifen metabolism requires further investigation.

Musculoskeletal symptoms were the most commonly reported side effect, affecting 40% of participants, consistent with prior reports [29,31]. Previous studies suggest an exposure-response relationship, whereby higher tamoxifen or active metabolite levels are associated with an increased occurrence of musculoskeletal symptoms [25,39]. In our cohort, carriers of the *UGT2B7* rs7439366 variant allele, particularly heterozygotes, showed a lower likelihood of musculoskeletal symptoms. The rs7439366 C allele has been associated with increased glucuronidation of tamoxifen metabolites [40,41,42], which may reduce systemic exposure to active metabolites and contribute to the observed protective effect. In addition, UGT2B has been shown to participate in the glucuronidation of bioactive lipid mediators implicated in pain and inflammation, including prostaglandins [43,44,45]. Enhanced UGT2B7 activity associated with this variant may therefore limit the accumulation of these mediators, reducing susceptibility to musculoskeletal symptoms, as observed in this study.

A similar protective trend was observed for *CYP3A4* rs2242480. Beyond its role in endoxifen formation, CYP3A4 catalyzes alternative tamoxifen metabolic pathways, including the formation of α-hydroxytamoxifen and N,N-didesmethyltamoxifen [11], which exhibit reduced or no anti-estrogenic activity. Notably, induction of CYP3A4 has been shown to reduce circulating levels of tamoxifen and its active metabolites [46,47,48], contrary to expectations based on increased metabolic activity. These observations suggest metabolic shunting away from endoxifen production toward alternative, less effective pathways. As rs2242480 has been associated with increased CYP3A4 enzymatic activity [49,50], this variant may similarly shift the balance of metabolite formation, reducing exposure to active metabolites and potentially explaining the lower frequency of musculoskeletal symptoms observed among carriers. Further functional and pharmacokinetic studies are required to validate this hypothesis.

Gynecological symptoms were reported by over 20% of participants, comparable to other cohorts [6,25]. Tamoxifen and its active metabolites act as selective estrogen receptor modulators, exerting both estrogen agonistic and antagonistic effects within the female genital tract [51]. The balance of these effects is influenced by menopausal status and tissue-specific estrogen receptor signaling [52,53]. In estrogen-responsive tissues such as the endometrium and cervicovaginal epithelium, partial agonist activity has been associated with proliferative and secretory changes [51,52], which may underlie gynecological symptoms including abnormal uterine bleeding and vaginal discharge.

Several studies have reported that gynecological side effects worsen with increasing endoxifen exposure and improve following tamoxifen dose reduction [25,39], suggesting a potential exposure-response relationship. However, dose-escalation studies have demonstrated that higher tamoxifen doses do not consistently translate into an increased gynecological symptom burden [54,55], underscoring the complexity of tamoxifen pharmacodynamics and interindividual variability in tissue sensitivity. In the present study, a protective effect against gynecological symptoms was observed among heterozygotes for *UGT2B15* rs4148269. This variant has been associated with increased glucuronidation of active tamoxifen metabolites [40], potentially reflecting enhanced UGT2B15 activity. Increased metabolite clearance may lead to lower circulating or tissue-level endoxifen exposure, thereby attenuating estrogen receptor-mediated effects in gynecological tissues and reducing symptom risk.

Conversely, heterozygotes for SULT1A2*2 and *SULT1E1* rs3736599 exhibited an increased likelihood of gynecological symptoms in this study. SULT1A2*2 has been associated with higher levels of tamoxifen active metabolites [56], whereas the functional impact of *SULT1E1* rs3736599 remains unclear. Given the role of SULT enzymes in tamoxifen metabolism [11], these observations are consistent with a pharmacokinetic trade-off, or survival paradox, whereby reduced sulfation capacity may prolong exposure to active tamoxifen metabolites, potentially enhancing therapeutic effect while increasing susceptibility to treatment-related toxicities. In contrast, higher SULT activity may accelerate metabolite clearance, which in theory could improve tolerability but compromise treatment efficacy. However, the role of SULT enzymes in tamoxifen response and breast cancer outcomes appears more complex, as reduced SULT activity has also been associated with poorer clinical outcomes in some studies [57,58,59]. This complexity underscores the need for further tamoxifen pharmacogenetic investigations that incorporate *SULT* variation, together with mechanistic studies to disentangle effects on efficacy and toxicity.

Hot flashes were common in our cohort, mirroring patterns observed in other studies [6,31,60]. This study did not observe any *CYP2D6*-associated pharmacogenetic effects, aligning with several previous investigations [6,27,29,60]. Similarly, one study examining variation in genes similar to those assessed here (*CYP2D6*, *CYP3A5*, *UGT1A4*, *UGT2B7*, and *UGT2B15*) also found no association with hot flashes, consistent with our findings [61]. In contrast, other studies have reported associations between *CYP2D6* genotype and hot flashes [25,62]. These discrepancies likely reflect differences in study design, population structure, allele frequencies, and the specific variants assessed. Variation in co-medication use may also contribute to inter-study differences. Co-administered medications that inhibit or induce drug-metabolizing enzymes can modify tamoxifen pharmacokinetics and the pattern of endoxifen formation [63], potentially shaping individual side-effect profiles. Notably, women with a history of smoking were more than twice as likely to experience hot flashes, a finding in line with previous literature [64,65,66].

This study presents its findings while acknowledging several limitations. Eligibility for the parent study required breast surgery, excluding non-surgical tamoxifen patients and introducing selection bias. The absence of associations surviving Bonferroni correction likely reflects limited statistical power rather than a definitive absence of effect. Given the exploratory nature of this study and the moderate-to-large effect sizes observed, these findings should be interpreted as hypothesis-generating and require replication in larger, prospectively designed African cohorts. Treatment adherence could not be reliably assessed, as the retrospective design provided only sporadic, self-reported information from physician notes. Reduced adherence could theoretically lower tamoxifen exposure and decrease reported side effects, thereby mimicking a protective genetic effect. However, the implicated variants affect drug metabolism and are not known to influence medication-taking behavior, and the observed associations were symptom-specific rather than generalized. Furthermore, in related analyses within this cohort, these variants were not associated with poorer disease-free survival, which would be expected under a sustained low-exposure model. In a prospective study, adherence could be more accurately monitored using patient diaries, pill counts, validated questionnaires, and pharmacokinetic measurements of tamoxifen and its metabolites, thereby enabling clearer differentiation between metabolic mechanisms and exposure-related effects. Symptom attribution is challenging, as observed effects may stem from natural physiological changes, such as aging or menopausal transition, or from co-administered medications, rather than tamoxifen alone. Despite these limitations, our findings provide preliminary evidence that phase II metabolism may influence tamoxifen tolerability. There is, however, a need to validate these findings by minimizing the indicated limitations.

In conclusion, this exploratory study highlights the high prevalence of treatment-related side effects among South African breast cancer patients and suggests that pharmacogenetic variation beyond *CYP2D6* may influence tamoxifen tolerability in African populations. These findings underscore the need for inclusive research that reflects Africa’s genetic diversity.

## Figures and Tables

**Table 1:** Summary of demographic and clinical characteristics of South African breast cancer patients receiving tamoxifen treatment.

Characteristic	Value
Ethnicity, n (%)	
Mixed	139 (83.7)
African	27 (16.3)
BMI (kg/m^2^), median (IQR)	29.5 (26.6–33.7)
Smoking history, n (%)	
Yes	65 (39.2)
No	87 (52.4)
Unknown	14 (8.4)
Co-medications, n (%)	
Yes	151 (91.0)
No	15 (9.0)
Menopausal status ^a^, n (%)	
Pre	66 (39.7)
Post	69 (41.6)
Unknown	31 (18.7)
Age at diagnosis (years), mean (±SD; range)	51.8 (10.3; 30–74)
Histological classification, n (%)	
IDC	148 (89.2)
ILC	9 (5.4)
Both	6 (3.6)
Unknown	3 (1.8)
Tumor grade ≥ G2, n (%)	
Yes	118 (71.1)
No	38 (22.9)
Unknown	10 (6.0)
Tumor stage ≥ T2, n (%)	
Yes	116 (69.9)
No	47 (28.3)
Unknown	3 (1.8)
Tamoxifen duration (months), median (IQR)	58 (41–61)
Reason for alternative endocrine therapy, n (%)	
Ineffective ^b^	33 (19.9)
Side effects	19 (11.4)
Unknown	3 (1.8)
No switch	111 (66.9)
Number of treatment-related side effects ^c^	
None	45 (27.1)
One	70 (42.2)
Two	35 (21.1)
Three or more	16 (9.6)

**Notes:** ^a^ Perimenopausal women were included in the premenopausal classification. ^b^ Ineffective tamoxifen treatment refers to disease progression or fear of disease progression, which resulted in a switch from tamoxifen to an alternative endocrine therapy. ^c^ Treatment-related side effects were defined as the occurrence of one or more of the following event categories: thromboembolic, endometrial, neurological, gynecological, vasomotor, or musculoskeletal. **Abbreviations:** BMI, body mass index; G2, tumor grade II; IDC, invasive ductal carcinoma; ILC, invasive lobular carcinoma; IQR, interquartile range; kg, kilograms; m^2^, square meters; SD, standard deviation; T2, tumor stage II.

**Table 2:** Logistic regression of demographic, clinical, and genetic factors associated with side effects, adjusted for ethnicity.

	No. of Patients	Univariate			Multivariate		
		OR	95% CI	*p*-Value	OR	95% CI	*p*-Value
**OVERALL SIDE EFFECTS**							
IDC							
No	11	1.00			1.00		
Yes	154	3.15	0.90–11.00	0.071	2.46	0.67–9.30	0.170
*SULT1A1* CNV ≤ 3							
No	51	1.00			1.00		
Yes	115	2.07	0.96–4.45	0.064	2.48	1.09–5.69	**0.030**
*SULT1E1* rs3736599							
G/G	108	1.00			1.00		
G/A	50	2.12	0.89–5.07	0.090	2.67	1.08–7.38	**0.042**
A/A	8	0.43	0.10–1.88	0.263	0.42	0.09–1.95	0.255
**MUSCULOSKELETAL SYMPTOMS**							
BMI		1.06	1.00–1.11	**0.047**	1.05	1.00–1.12	0.063
*CYP3A4* rs2242480							
G/G	35	1.00			1.00		
G/A	78	0.44	0.19–0.99	**0.049**	0.35	0.12–1.00	0.051
A/A	53	0.61	0.24–1.53	0.288	0.49	0.15–1.55	0.226
*UGT2B7* rs7439366							
T/T	19	1.00			1.00		
T/C	65	0.27	0.09–0.78	**0.016**	0.26	0.07–0.93	**0.040**
C/C	82	0.54	0.20–1.51	0.241	0.52	0.15–1.70	0.286
**VASOMOTOR SYMPTOMS**							
BMI		0.93	0.88–0.99	**0.029**	0.95	0.89–1.01	0.144
Smoking history							
No	87	1.00			1.00		
Yes	65	1.98	0.98–4.02	0.058	2.74	1.12–6.95	**0.029**
*UGT1A4* rs11888492							
C/C	81	1.00			1.00		
C/G	70	0.81	0.41–1.63	0.560	0.61	0.24–1.50	0.290
G/G	15	0.24	0.05–1.25	0.091	0.28	0.04–1.40	0.158
*SULT1A1* rs4149393 ^a^							
A/A	74	1.00			1.00		
A/G	87	0.83	0.42–1.62	0.580	0.87	0.38–1.97	0.734
G/G	5	7.63	0.80–72.42	0.077	-	-	-
**GYNECOLOGICAL SYMPTOMS**							
*UGT2B15* rs4148269							
A/A	72	1.00			1.00		
A/C	69	0.40	0.16–0.97	**0.043**	0.37	0.14–0.93	**0.039**
C/C	25	1.04	0.36–2.94	0.947	1.41	0.45–4.22	0.545
SULT1A2*2 ^b^							
*1/*1	86	1.00			1.00		
*1/*2	70	2.20	1.02–4.75	**0.044**	2.22	1.01–5.05	**0.050**
*2/*2	10	0.58	0.07–4.97	0.620	0.36	0.02–2.39	0.366
*SULT1E1* rs3736599							
G/G	108	1.00			1.00		
G/A	50	2.32	1.05–5.09	**0.037**	2.87	1.22–6.91	**0.016**
A/A	8	1.67	0.31–8.94	0.550	1.99	0.27–10.19	0.437

**Notes:** Variables with *p*-values ≤ 0.1 in univariate analyses were included in the multivariate model. *p*-values ≤ 0.05 are indicated in bold. ^a^ *SULT1A1*: rs4149393 was included in the model as a representative marker for rs4149393, rs4149394, and rs1042157 due to strong linkage disequilibrium among the variants (D′ = 1; R^2^ = 0.9). ^b^ The SULT1A2*2 haplotype is defined by the rs1059491 and rs1136703 variants which are in perfect linkage disequilibrium (D′ = 1; R^2^ = 1). **Abbreviations:** BMI, body mass index; CI, confidence interval; CNV, copy number variation; IDC, invasive ductal carcinoma; OR, odds ratio; T2, tumor stage II.

## Data Availability

The data that support the findings of this study are available from the corresponding author [C.D.] upon reasonable request.

## Supplementary Materials

The following supporting information can be downloaded at https://www.mdpi.com/article/10.3390/genes17030252/s1. Figure S1: Distribution of *SULT1A1* copy number among South African breast cancer patients; Table S1: Univariate analysis of CYP2D6-predicted phenotype in relation to treatment-related side effects, adjusted for ethnicity.
