# Supplementary material for: SULT and UGT Genetic Variants Modulate Side Effect Profiles in South African Breast Cancer Patients Treated with Tamoxifen
Source: Genes (Basel). 2026 Feb 24;17(3):252. doi: 10.3390/genes17030252 (PMC13025226; doi:10.3390/genes17030252)
Supplement: Supplementary file 1 [file genes-17-00252-s001.zip › Supplementary Materials_Genes-1.pdf]

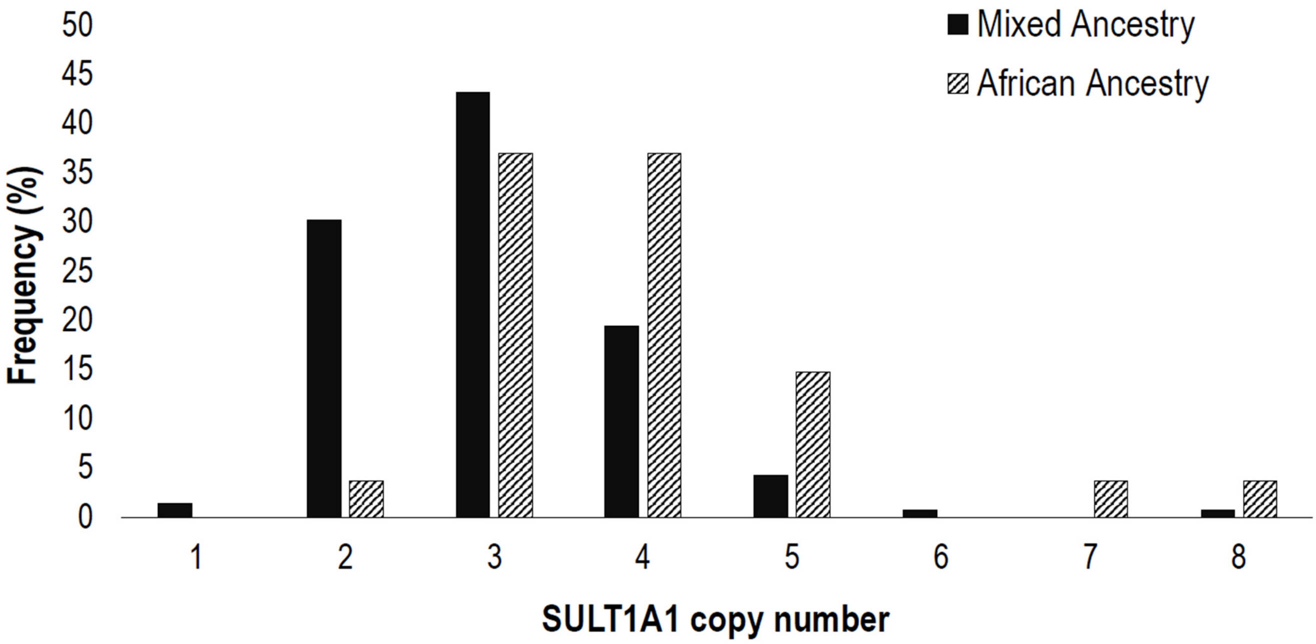

Figure S1. Distribution of *SULT1A1* copy number among South African breast cancer patients. Copy number frequencies (%) are shown separately for participants of Mixed Ancestry (solid black bars) and African Ancestry (striped bars).
