# Supplementary material for: SULT and UGT Genetic Variants Modulate Side Effect Profiles in South African Breast Cancer Patients Treated with Tamoxifen
Source: Genes (Basel). 2026 Feb 24;17(3):252. doi: 10.3390/genes17030252 (PMC13025226; doi:10.3390/genes17030252)
Supplement: Supplementary file 1 [file genes-17-00252-s001.zip › Supplementary Materials_Genes-2.pdf]

Table S1. Univariate analysis of CYP2D6 predicted phenotype in relation to treatment-related side effects, adjusted for ethnicity.

	No. of patients	Overall side effects			Musculoskeletal			Vasomotor			Gynecological		
		**OR**	**95% CI**	**P-value**	**OR**	**95% CI**	**P-value**	**OR**	**95% CI**	**P-value**	**OR**	**95% CI**	**P-value**
EM	88	1.00			1.00			1.00			1.00		
UM	2	0.39	0.02-6.52	0.512	1.61	0.10-26.67	0.740	-	-	-	-	-	-
IM	52	0.57	0.26-1.23	0.151	0.71	0.35-1.48	0.364	0.68	0.32-1.45	0.323	1.00	0.44-2.27	0.996
PM	7	0.98	0.18-5.41	0.977	0.64	0.12-3.52	0.611	1.35	0.28-6.48	0.705	1.41	0.25-7.92	0.693
UM + EM + IM	142	1.00			1.00			1.00			1.00		
PM	7	1.22	0.23-6.58	0.817	0.71	0.13-3.84	0.695	1.58	0.34-7.40	0.563	1.45	0.26-7.90	0.671

Abbreviations: CI, confidence interval; EM, extensive metabolizer; IM, intermediate metabolizer; OR, odds ratio; PM, poor metabolizer; UM, ultrarapid metabolizer
